# IRF3 and IRF7 mediate neovascularization via inflammatory cytokines

**DOI:** 10.1111/jcmm.14247

**Published:** 2019-04-01

**Authors:** Karin H. Simons, Margreet R. de Vries, Rob C. M. de Jong, Hendrika A. B. Peters, J. Wouter Jukema, Paul H. A. Quax

**Affiliations:** ^1^ Department of Surgery Leiden University Medical Center Leiden The Netherlands; ^2^ Einthoven Laboratory for Experimental Vascular Medicine Leiden University Medical Center Leiden The Netherlands; ^3^ Department of Cardiology Leiden University Medical Center Leiden The Netherlands

**Keywords:** angiogenesis, arteriogenesis, inflammation, interferon regulatory factor, neovascularization

## Abstract

**Objective:**

To elucidate the role of interferon regulatory factor (IRF)3 and IRF7 in neovascularization.

**Methods:**

Unilateral hind limb ischaemia was induced in *Irf3^−/−^, Irf7^−/−^* and C57BL/6 mice by ligation of the left common femoral artery. Post‐ischaemic blood flow recovery in the paw was measured with laser Doppler perfusion imaging. Soleus, adductor and gastrocnemius muscles were harvested to investigate angiogenesis and arteriogenesis and inflammation.

**Results:**

Post‐ischaemic blood flow recovery was decreased in *Irf3^−/−^*and *Irf7^−/−^* mice compared to C57BL/6 mice at all time points up to and including sacrifice, 28 days after surgery (t28). This was supported by a decrease in angiogenesis and arteriogenesis in soleus and adductor muscles of *Irf3^−/−^* and *Irf7^−/−^* mice at t28. Furthermore, the number of macrophages around arterioles in adductor muscles was decreased in *Irf3^−/−^*and *Irf7^−/−^* mice at t28. In addition, mRNA expression levels of pro‐inflammatory cytokines (*tnfα*, *il6*, *ccl2*) and growth factor receptor (*vegfr2*), were decreased in gastrocnemius muscles of *Irf3^−/−^* and *Irf7^−/−^* mice compared to C57BL/6 mice.

**Conclusion:**

Deficiency of IRF3 and IRF7 results in impaired post‐ischaemic blood flow recovery caused by attenuated angiogenesis and arteriogenesis linked to a lack of inflammatory components in ischaemic tissue. Therefore, IRF3 and IRF7 are essential regulators of neovascularization.

## 1 INTRODUCTION

Occlusive arterial disease is the narrowing of an artery, resulting in impaired blood flow and ischaemia distal to the obstruction. To prevent or resolve ischaemia, neovascularization is induced spontaneously to restore blood flow. Angiogenesis and arteriogenesis are the main components of neovascularization and are induced under pathological conditions, such as peripheral arterial disease and myocardial infarction.

Angiogenesis, the sprouting of new capillaries from pre‐existing vasculature,^1^ responds to hypoxia by up‐regulating pro‐angiogenic growth factors such as vascular endothelial growth factor (VEGF).^2^ These growth factors induce endothelial cells from the pre‐existing vasculature to migrate, grow and differentiate to shape new capillaries.^3^, ^4^ Arteriogenesis, the maturation of pre‐existing collaterals into arterioles,^5^, ^6^, ^7^, ^8^ is induced by inflammation, shear stress and circumferential stretch on the vascular wall after occlusion, mainly proximal to the occlusion. Inflammatory cells such as macrophages, monocytes, but also CD4+ and CD8+ T cells adhere and invade into the vascular wall and produce inflammatory cytokines, chemokines and growth factors, which cause further maturation of arterioles and induce angiogenesis. Subsequently, smooth muscle cells proliferate and migrate towards the vessel wall, resulting in full functional arterioles.^7^, ^9^, ^10^ Both angiogenesis and arteriogenesis are essential for accurate neovascularization, to restore blood flow after arterial occlusions.

For the production of inflammatory cytokines, chemokines and growth factors, inflammatory cells require activation. Substantial activators are pattern recognition receptors, such as toll‐like receptors (TLR).^11^, ^12^ TLR3 expressed in intracellular vesicles recognizes dsRNA, which activates a Toll/interleukin‐1 receptor domain‐containing adapter‐inducing interferon‐β protein (TRIF)‐dependent pathway. The TLR3‐TRIF pathway induces type‐I IFN production, via interferon regulatory factor (IRF)3 and IRF7 phosphorylation, and pro‐inflammatory cytokine production via transcription factors nuclear factor kappa‐light‐chain‐enhancer of activated B cells (NFĸB).^12^, ^13^, ^14^, ^15^ IRF3 and IRF7 are mainly phosphorylated downstream TLR3, but might also be partly activated via other TLRs (eg TLR4, TLR7, TLR9).^12^, ^15^ It is assumed that NFĸB activation results in pro‐inflammatory cytokine production and IRF3/IRF7 phosphorylation results in type‐I IFN production. However, it is observed that TLR3 can also activate pro‐inflammatory transcription factors via IRF3 and IRF7 and may be potential regulators of cell proliferation and survival.^16^, ^17^, ^18^, ^19^ Since neovascularization is mainly induced by pro‐inflammatory cytokines and growth factors, we hypothesize an important role for IRF3 and IRF7 in arteriogenesis and angiogenesis.

In the current study, we aimed to elucidate the role of IRF3 and IRF7 in vivo in post‐ischaemic neovascularization in a hind limb ischia (HLI) mouse model, using wild‐type C57BL/6*, Irf3^−/−^*and *Irf7^−/−^* mice.

## 2 MATERIAL AND METHODS

### 2.1 Mice

This study was performed in compliance with the Dutch government guidelines and the Directive 2010/63/EU of the European Parliament. All experiments were approved by the committee on animal welfare of the Leiden University Medical Center (Leiden, the Netherlands). For the experiments 10‐18‐week‐old male mice were used. C57BL/6 mice were purchased from Charles River Laboratories and Irf3*^−/−^* and Irf7*^−/−^* mice were kindly provided by Dr Taniguchi (University of Tokyo, Japan) and bred in our facility.^20^ Mice were fed a chow diet ad libitum.

### 2.2 Murine HLI model

Mice were anaesthetized with an intraperitoneal injection of midazolam (8 mg/kg; Roche Diagnostics), medetomidine (0.4 mg/kg; Orion), and fentanyl (0.08 mg/kg; Janssen Pharmaceuticals). Unilateral hind limb ischaemia (HLI) was performed. In brief, a single ligation model was performed by electrocoagulation of the left common femoral artery proximal to the bifurcation of the popliteal and saphenous artery.^21^, ^22^ After surgery, anaesthesia was antagonized with flumazenil (0.7 mg/kg, Fresenius Kabi). Buprenorphine (0.1 mg/kg, MSD Animal Health) was given after surgery to relieve pain in a fixed regime and when circumstances required this was repeated. Number of total operated mice was 11 per group, however, in the C57BL/6 group 5 mice deceased during or after surgery and 2 and 4 mice, respectively in the *Irf3^−/−^* and *Irf7^−/−^* group. Mice characteristics of *Irf3^−/−^*, *Irf7^−/−^* and C57BL/6 mice are shown in Table S1. Body weight at day of surgery (*t* = 0) and when killed (*t* = 28) was similar in the *Irf3^−/−^* and *Irf7^−/−^* mice compared to C57BL/6 mice.

### 2.3 Laser Doppler perfusion imaging

Post‐ischaemic blood flow recovery was measured in the left ischaemic and right non‐ischaemic paw of *Irf3^−/−^*, *Irf7^−/−^* and C57BL/6 mice with the use of laser Doppler perfusion imaging (LDPI) (Moor Instruments).^23^ Blood flow was measured before and directly after surgery and at day 3, 6, 9, 14, 21 and 28. Paw perfusion was expressed as a ratio of left ischaemic to right non‐ischaemic paw.^24^ Before LDPI, mice were anaesthetized as described before^25^, ^26^ with an intraperitoneal injection of midazolam (8 mg/kg) and medetomidine (0.4 mg/kg). After LDPI, anaesthesia was antagonized by subcutaneous injection of flumazenil (0.7 mg/kg). Mice were killed 28 days after surgery after the last LDPI via cervical dislocation after subcutaneous admission of analgesic fentanyl (0.08 mg/kg). The adductor, soleus and gastrocnemius muscles were harvested for either (immuno) histochemical analysis or RT‐qPCR.

### 2.4 Immunohistochemistry

Adductor muscles were fixed in 4% formaldehyde and embedded in paraffin, soleus and gastrocnemius muscles were snap frozen on dry ice and stored at −80°C.

Cross sections of 6 μm were made throughout the embedded adductor muscle. Adductor muscle sections were stained with alpha smooth muscle cell actin (aSMActin, DAKO) to visualize vascular smooth muscle cells (VSMC). Stained slides were photographed (20× magnification) with microscope photography software (Axiovision, Zeiss) and analysed with ImageJ (FIJI) by counting the number of arterioles and measuring the diameters of each arteriole with a visible lumen to determine arteriogenesis.^25^ In addition, adductor muscle sections were stained with alpha smooth muscle cell actin (pink) and MAC3 (green) (BD Pharmingen) to visualize macrophages around arterioles. Images were acquired on a Philips Ultra Fast Slide Scanner (Philips Digital Pathology Solutions, Best) and analysed by counting the number of macrophages around the arterioles divided by the circumference of the arteriole (presented as macrophages/μm). However, 12 sections, in the same area in all mice were analysed per mice per leg and the average was used for analyses. Negative controls were performed by omitting either the secondary antibody of aSMActin or MAC3, or PBS.

Fresh‐frozen soleus muscles were cross sectioned in 6 μm slices with a cryostat and sections were fixated in acetone. To visualize endothelial cells, soleus muscle sections were stained with CD31 (BD Pharmingen). Stained slides were photographed (20× magnification) with microscope photography software. The number of CD31+ structures was analysed with ImageJ, which was used to determine angiogenesis and was presented in absolute numbers of CD31+ structures per area.

### 2.5 RNA isolation, cDNA synthesis and RT‐PCR from adductor muscles

RNA was isolated from snap frozen gastrocnemius muscles 28 days after surgery as described before.^27^ In brief, muscle tissues were crushed with mortar and pestle, while using liquid nitrogen to preserve sample integrity. RNA was isolated according to manufacturer's protocol using TRIzol Reagent (Life Technologies) (FFPE RNA isolation kit, Qiagen), after which sample concentration and purity were examined by nanodrop (Nanodrop Technologies). Complementary DNA (cDNA) was synthesized using a High‐Capacity cDNA Reverse Transcription Kit according to the manufacturer's protocol (Applied Biosystems). To measure the expression of *tnfα *(forward TGAACTTCGGGGTGATCGG, reverse CTCCTCCACTTGGTGGTTTG), *ccl2 *(forward TATTGGCTGGACCAGATGCG, reverse GGACACTGGCTGCTTGTGAT), *il6 *(forward TCCGGAGAGGAGACTTCACA, reverseTTGCCATTGCACAACTCTTTTC), *il1β *(forward AGCTTCCTTGTGCAAGTGTC, reverse TGGGGTCCGTCAACTTCAAA), *sdf1 *(forward TTTCACTCTCGGTCCACCTC, reverse AGCTCAGGCTGACTGGTTTAC), *vegf164 *(forward AACGATGAAGCCCTGGAGTG, reverse GACAAACAAATGCTTTCTCCG), *vegfr1 *(forward GTCTCTTCGCGGTTAGCTCC, reverse AAAAGAAGCCCAGAGAGAGGT) and *vegfr2* (forward CGTTAAGCGGGCCAATGAAG, reverse CTAGTTTCAGCCGGTCCCTG), RT‐qPCR was performed using QuantiTect SYBR Green PCR Kit (Qiagen). GAPDH (Applied Biosystems) was used as a housekeeping gene. All RT‐qPCRs were performed on a 7500/7500 Fast Real‐Time PCR System (Applied Biosystems) and the 2‐ΔΔCt method was used to analyse the relative changes in gene expression.

### 2.6 Statistical analysis

All data are presented as mean ± SEM. In statistics software GraphPad Prism 7.0, statistical analyses on parametric data were performed by using a 2‐tailed Student's *t* test to compare individual groups, Mann‐Whitney test was used for nonparametric data. A 1‐way ANOVA was performed on parametric data comparing more than two groups and a Kruskal‐Wallis test was performed on nonparametric data, followed by Tukey's post hoc test. P value of <0.05 was considered significant.

## 3 RESULTS

### 3.1 Impact of IRF3 and IRF7 on post‐ischaemic blood flow recovery

**Figure 1:**
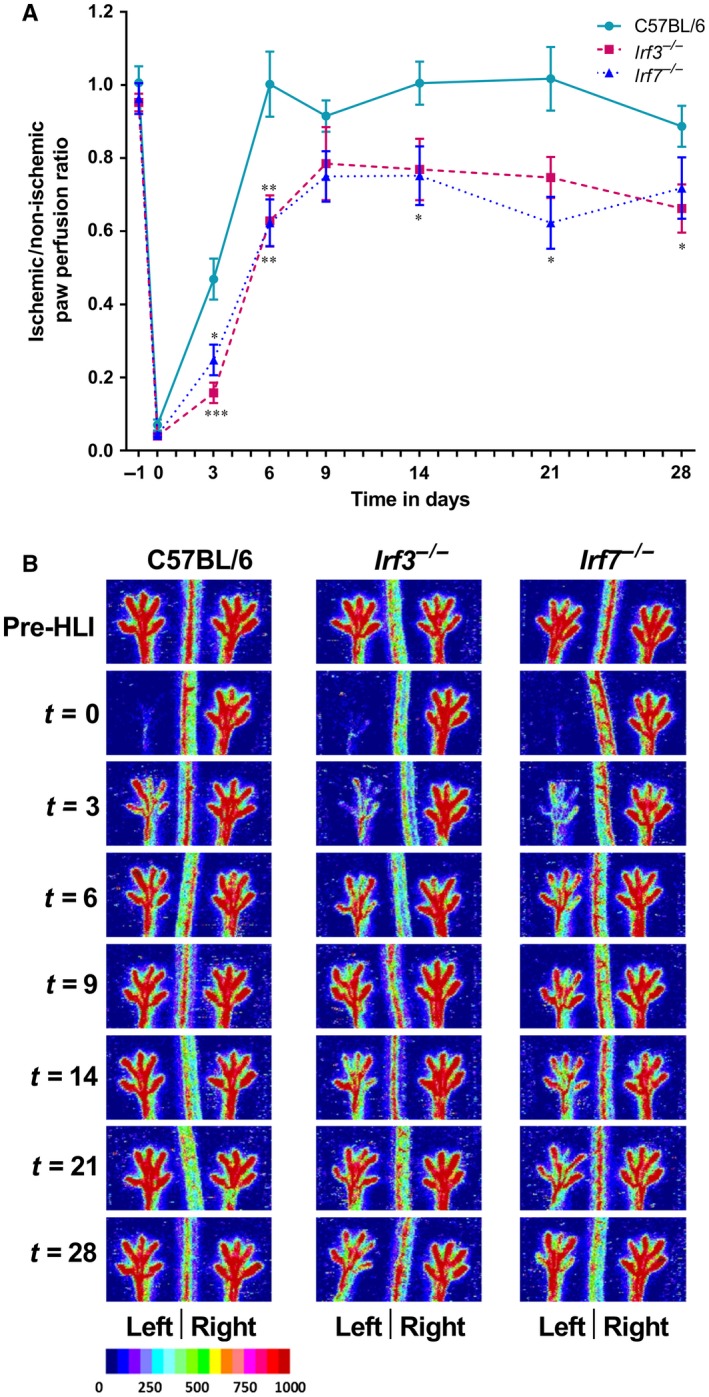
Post‐ischaemic blood flow recovery Laser Doppler perfusion imaging was used to measure paw perfusion in *Irf3* ^−/−^, *Irf7* ^−/−^ and C57BL/6 mice. Paw perfusion was measured in the left and right paw before ligation of the left femoral, and 3, 6, 9, 14, 21 and 28 days after ligation. (A) Paw perfusion is expressed as a ratio of the left ischaemic to right non‐ischaemic (L/R) paw perfusion. (B) Representative pictures of the left and right paw before ligation of the left femoral, and 3, 6, 9, 14, 21 and 28 days after ligation of *Irf3* ^−/−^, *Irf7* ^−/−^ and C57BL/6 mice are shown. Data is presented as mean SEM; *P < 0.05, **P < 0.01, ***P < 0.001, Mann Whitney test was used. C57BL/6 n = 6, *Irf3* ^−/−^ n = 9, *Irf7* ^−/−^ n=7.

To study the role of IRF3 and IRF7 in post‐ischaemic blood flow recovery, we ligated the left femoral artery in C57BL/6, *Irf3^−/−^* and *Irf7^−/−^*mice and analysed paw perfusion before ligation and 3, 6, 9, 14, 21 and 28 days after ligation. Results of the LDPI, expressed as the left ischaemic/right non‐ischaemic ratio, are shown in Figure 1. After ligation, the paw perfusion decreased tremendously in all groups to ratios of approximately 0.05. The fact that these ratios are similar in all groups, suggests that the number of pre‐existing collaterals, that allow residual blood flow to the paw after ligation, is similar. C57BL/6 mice already recovered almost 50% 3 days after surgery, whereas the *Irf3^−/−^* (*P *= 0.0005) and *Irf7^−/−^*(*P *= 0.01) mice only recovered 20%. Both *Irf3^−/−^* (*P *= 0.006) and *Irf7^−/−^* (*P *= 0.006) mice also showed an impaired blood flow recovery compared to C57BL/6 mice 6 days after ligation. C57BL/6 mice already reached their maximal recovery 6 days after surgery, showing a similar paw perfusion as before the ligation, which did not change until sacrifice. After 9 days, *Irf3^−/−^* and *Irf7^−/−^* mice reached their maximal recovery of, respectively, 78% and 75% which did not further increase up to the time of sacrifice, 4 weeks after surgery. Both *Irf3^−/−^* and *Irf7^−/−^* mice did not manage to reach the same level of post‐ischaemic blood flow recovery as C57BL/6 mice. This indicates that IRF3 and IRF7 have an important role in post‐ischaemic blood flow recovery.

### 3.2 Angiogenesis in soleus muscles

**Figure 2:**
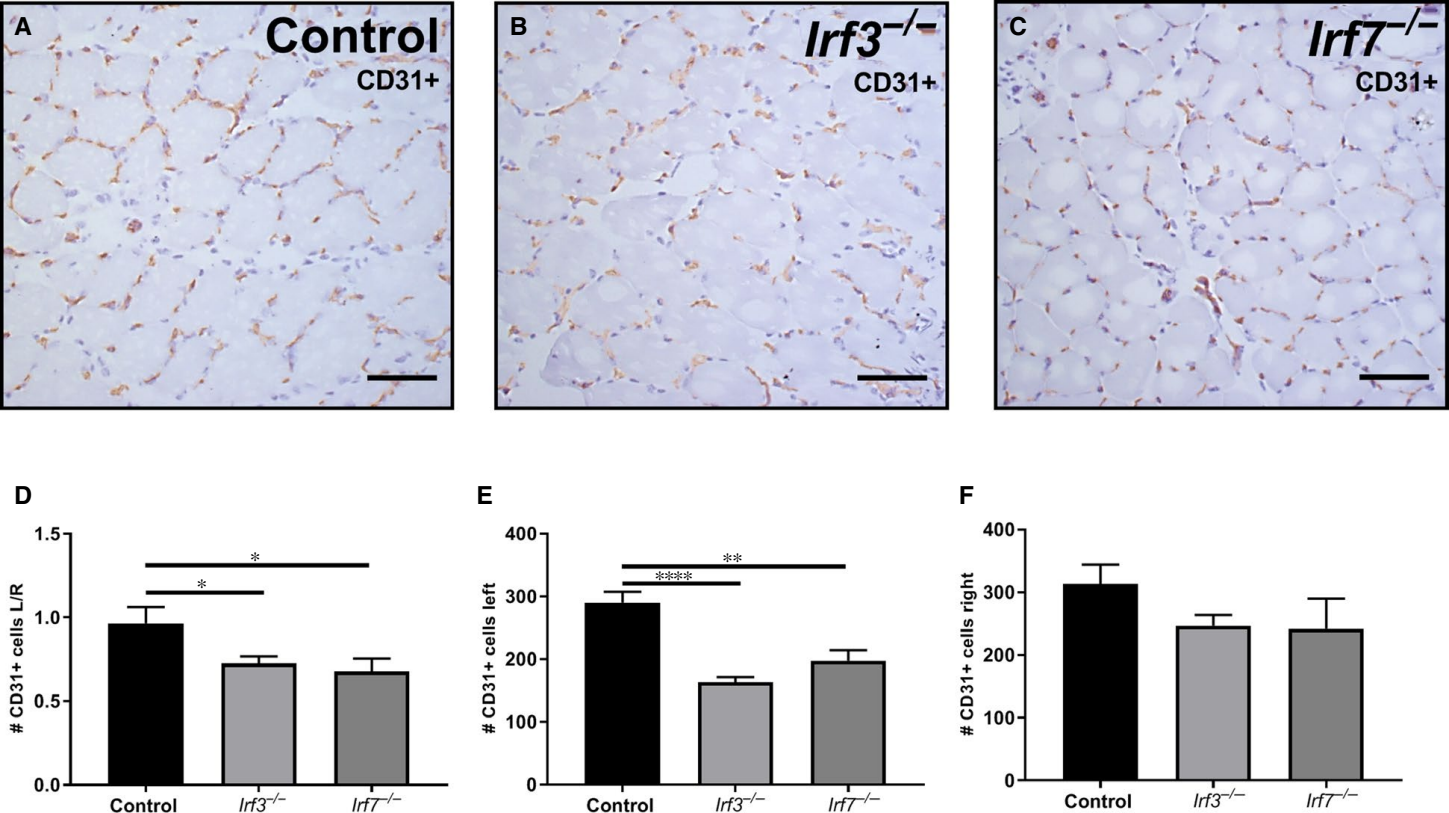
Angiogenesis in CD31+ stained soleus muscles. Representative image of the left ischaemic soleus muscle stained with CD31 (20× magnification) of (A) C57BL/6 mice (B) *Irf3^−/−^*mice and (C) *Irf7^−/−^* mice sacrificed 28 days after surgery (D) Ratio of the number of CD31+ cells in the soleus muscle of left ischaemic/right non‐ischaemic is shown. (E) The number of CD31+ cells in the soleus muscle of the left ischaemic calf is shown. (F) The number of CD31+ cells in the soleus muscle of the right non‐ischaemic calf is shown. Data is presented as mean SEM; *P < 0.05; **P < 0.01, ****P < 0.0001, a 2‐tailed Student's *t* test was used. C57BL/6 n = 6, *Irf3^−/−^* n = 9, *Irf7^−/−^* n = 6

To investigate the underlying cause of the reduced post‐ischaemic blood flow recovery in *Irf3^−/−^* and *Irf7^−/−^* mice, we determined the angiogenic capillary formation in the soleus muscles of *Irf3^−/−^*, *Irf7^−/−^* and C57BL/6 mice. Soleus muscles of the left ischaemic and right non‐ischaemic calf were stained with CD31 (Figure 2A‐C). The number of CD31+ cells, expressed as left ischaemic/right non‐ischaemic, in the soleus muscle of *Irf3^−/−^* mice (*P *= 0.02) and *Irf7^−/−^* mice (*P *= 0.04) was decreased compared to C57BL/6 mice 28 days after surgery (Figure 2D). This suggests a decrease in angiogenesis. The number of CD31+ cells in the left ischaemic calf was significantly decreased in the soleus muscle of both *Irf3^−/−^* (*P* < 0.0001) and *Irf7^−/−^*(*P *= 0.003) mice compared to C57BL/6 mice (Figure 2E). The right non‐ischaemic calf showed no differences in number of CD31+ cells (Figure 2F) in *Irf3^−/−^* and *Irf7^−/−^* compared to C57BL/6 mice, indicating that there is no genetic difference in angiogenesis between *Irf3^−/−^*, *Irf7^−/−^* and C57BL/6 mice.

### 3.3 Arteriogenesis in adductor muscles

**Figure 3:**
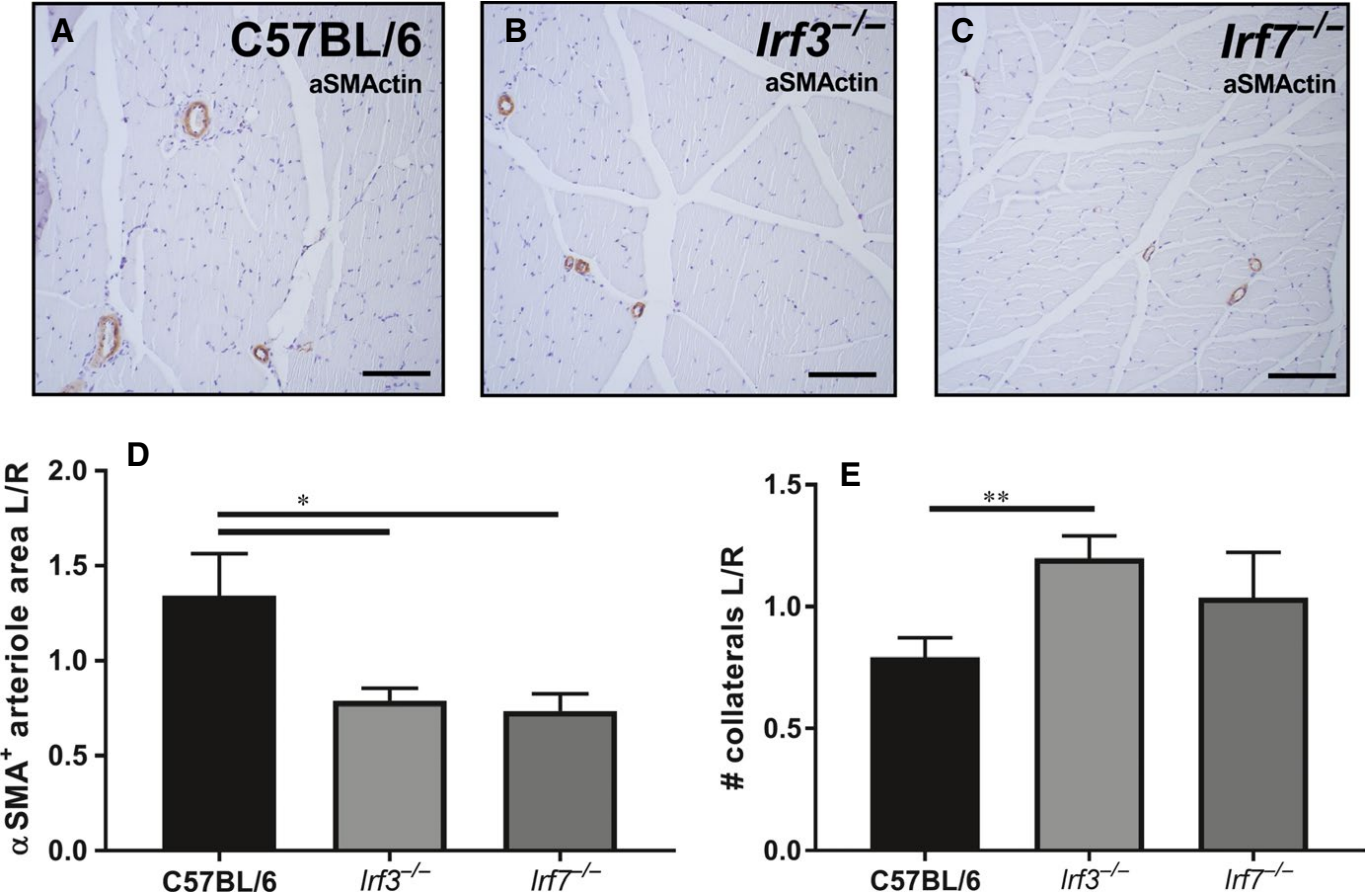
Arteriogenesis in aSMActin stained adductor muscles. (A) Representative image of the left ischaemic adductor muscle of C57BL/6 mice sacrificed 28 days after surgery, stained with aSMActin (20× magnification) and (B) *Irf3^−/−^* mice and (C) *Irf7^−/−^* mice. (D) Left ischaemic/right non‐ischaemic ratio is shown of the αASMActin positive arteriole area (µm^2^). (E) Left ischaemic/right non‐ischaemic ratio is shown of the number of arterioles. Data is presented as mean SEM; *P < 0.05; **P < 0.01, a 2‐tailed Student's *t* test was used. C57BL/6 n = 6, *Irf3^−/−^* and *Irf7^−/−^* n = 7

In addition to angiogenesis, arteriogenesis also is an important process during neovascularization. To investigate arteriogenesis, adductor muscles were stained with aSMActin (Figure 3A‐C) and the diameter of arterioles were measured (Figure S1A,B) together with the number of arterioles. The diameter of the aSMActin+arterioles, expressed as left ischaemic/right non‐ischaemic, was smaller in *Irf3^−/−^* mice (*P *= 0.04) and *Irf7^−/−^* mice (*P *= 0.02) compared to C57BL/6 mice 28 days after surgery (Figure 3D).The diameter of the aSMActin + arterioles was decreased in the individual left ischaemic adductor muscles of *Irf3^−/−^* (*P *= 0.005) and *Irf7^−/−^* (*P *= 0.02) mice compared to C57BL/6 mice (Figure S1C). No significant differences were observed in the diameter of the aSMActin + arterioles of the right non‐ischaemic adductor muscle of C57BL/6 compared to *Irf3^−/−^* and *Irf7^−/−^* mice. (Figure S1D). The number of arterioles was increased in the adductor muscles of *Irf3^−/−^* mice (*P *= 0.007) after 28 days compared to C57BL/6 mice but was not significantly different in *Irf7^−/−^*mice compared to C57BL/6 mice adductor muscles (Figure 3E). Interestingly, the number of arterioles was similar in adductor muscles of *Irf3^−/−^*, *Irf7^−/−^* and C57BL/6 mice in both the individual left ischaemic and right non‐ischaemic muscle, indicating no differences in the number of pre‐existing collaterals (Figure S1E,F). In conclusion, these results suggest an important role for IRF3 and IRF7 in arteriogenesis.

### 3.4 Perivascular accumulation of inflammatory cells

**Figure 4:**
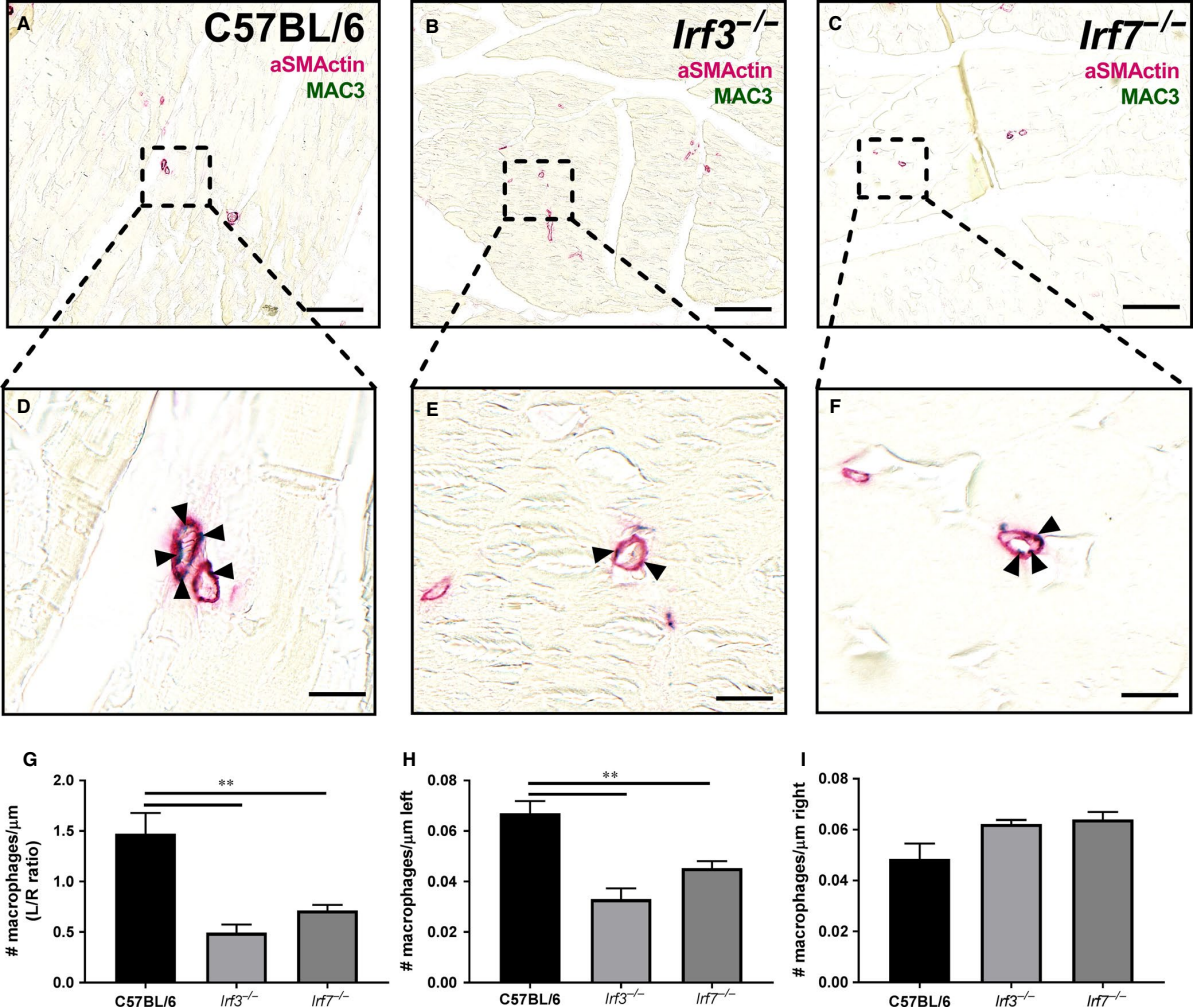
Macrophages around arterioles. An immunohistochemical double staining was performed on adductor muscles of *Irf3^−/−^*, *Irf7^−/−^* and C57BL/6 mice sacrificed 28 days after HLI. aSMActin (pink) was used to show the arterioles and MAC3 (green) to show the macrophages around the collaterals. Representative images of the left ischemic adductor muscle of (A) C57BL/6 mice, (B) *Irf3^−/−^* mice and (C) *Irf7^−/−^* mice stained with aSMActin and MAC3 is shown (scalebar = 100 μm) and a zoom in of the arterioles of the concerned (D) C57BL/6 mice, (E) *Irf3^−/−^* mice, (F) *Irf7^−/−^* mice adductor muscle is shown (scalebar = 10 µm). Arrows indicate were the macrophages are located in the arterioles. (G) Ratio (left ischemic/right non‐ischemic) of the number of macrophages per μm circumference of arterioles in the adductor muscle is shown. (H) Number of macrophages per μm circumference of arterioles in the left ischemic adductor muscle is shown (I) Number of macrophages per μm circumference of arterioles in the right non‐ischemic adductor muscle is shown. Data is presented as mean SEM; **P < 0.01, Mann‐Whitney test was used. C57BL/6 n = 5, *Irf3^−/−^* n = 7, *Irf7^−/−^* n = 7

Perivascular inflammation was demonstrated by the number of macrophages around the arterioles. An immunohistochemical double staining was performed on adductor muscles of *Irf3^−/−^*, *Irf7^−/−^* and C57BL/6 mice killed 28 days after HLI. Here, aSMActin was used to show the arterioles and MAC3 to show the macrophages around the collaterals (Figures 4A‐F and S2). The number of macrophages per μm circumference, expressed as left ischaemic/right non‐ischaemic, in the adductor muscle of *Irf3^−/−^* mice (*P *= 0.003) and *Irf7^−/−^* mice (*P *= 0.003) compared to C57BL/6 mice 28 days after surgery was decreased (Figure 4G). The number of macrophages per μm circumference in the left ischaemic adductor muscle was also significantly decreased in both *Irf3^−/−^* (*P *= 0.002) and *Irf7^−/−^*(*P *= 0.005) mice compared to C57BL/6 mice (Figure 4H). In the right non‐ischaemic adductor muscle, *Irf3^−/−^* and *Irf7^−/−^* mice showed no differences in macrophages per μm circumference compared to C57BL/6 mice (Figure 4I).

### 3.5 mRNA expression of essential genes for arteriogenesis and angiogenesis

**Figure 5:**
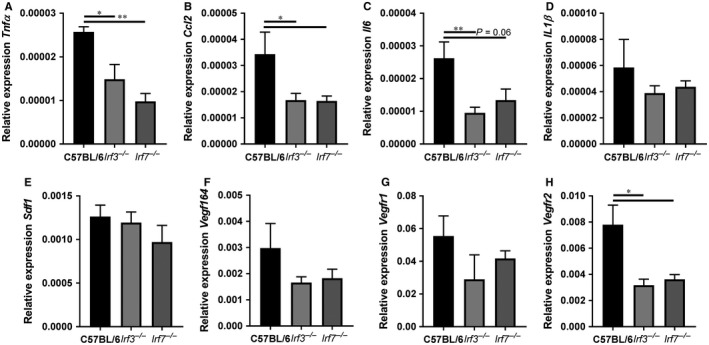
mRNA expression of essential genes for arteriogenesis and angiogenesis. RNA was isolated of gastrocnemius muscles of *Irf3^−/−^*, *Irf7^−/−^* and C57BL/6 mice sacrificed 28 days after HLI, and used for RT‐qPCR analysis. Relative mRNA expression is shown of (A) *tnfα*, (B) *ccl2*, (C) *il6*, (D) *il1β*, (E) *sdf1*, (F) *vegf164*, (G) *vegfr1*, and (H) *vegr2*. Relative expression of mRNA is shown to GAPDH of the left ischemic gastrocnemius muscle. Data is presented as mean SEM; *P < 0.05; **P < 0.01, a 1‐way ANOVA and a Kruskal‐Wallis test was used. n = 5‐8.

RNA from the gastrocnemius muscles was isolated and the mRNA expression of *tnfα*, *ccl2*, *il6*, *il1β*, *sdf1*, *vegf164*, *vegfr1* and *vegfr2* was measured with qPCR as genes essential for angiogenesis and arteriogenesis. The relative mRNA expression of inflammatory cytokines *tnfα*, *ccl2* and *il6* was decreased in left ischaemic gastrocnemius muscles of *Irf3^−/−^* and *Irf7^−/−^* mice compared to C57BL/6 mice (Figure 5A‐C). However, *Il1β* and *sdf1* were not differentially expressed in gastrocnemius muscles of *Irf3^−/−^,* *Irf7^−/−^* and C57BL/6 mice (Figure 5D,E). However, VEGF and the first receptor (VEGFR1) showed a trend towards a down‐regulation in gastrocnemius muscles of *Irf3^−/−^*and *Irf7^−/−^* mice compared to C57BL/6 mice (Figure 5F,G). Interestingly, the mRNA expression levels of the second receptor (VEGFR2) were decreased in the left ischaemic gastrocnemius muscles of *Irf3^−/−^* and *Irf7^−/−^* mice compared to C57BL/6 mice (Figure 5H). No differences were observed in the mRNA expression of *tnfα*, *ccl2*, *il6*, *il1β*, *sdf1*, *vegf164*, *vegfr1* and *vegfr2* in the right non‐ischaemic gastrocnemius muscles of *Irf3^−/−^* and *Irf7^−/−^* mice compared to control mice (Figure S3). These results demonstrate that IRF3 and IRF7 regulate the expression of several genes essential for angiogenesis and arteriogenesis.

## 4 DISCUSSION

In the present study, we observed a decreased post‐ischaemic blood flow recovery in *Irf3^−/−^* and *Irf7^−/−^* mice compared to C57BL/6 mice, using a HLI model. This was supported by a decrease in angiogenesis and arteriogenesis in, respectively, soleus and adductor muscles of the *Irf3^−/−^* and *Irf7^−/−^* mice. Furthermore, mRNA expression levels of several pro‐inflammatory cytokines (*tnfα*, *il6*, *ccl2*) and growth factor receptor (*vegfr2*) that are essential for the induction of angiogenesis and arteriogenesis, were decreased in the gastrocnemius muscles of *Irf3^−/−^* and *Irf7^−/−^* mice. This indicates a decreased post‐ischaemic blood flow recovery caused by an impairment in angiogenesis and arteriogenesis due to lack of inflammatory components in the ischaemic tissue.

Angiogenesis provides a better distribution of blood to ischaemic tissue. Since we observed a decreased post‐ischaemic blood flow recovery in the paw of *Irf3^−/−^* and *Irf7^−/−^* mice compared to C57BL/6 mice, we hypothesized attenuated angiogenesis in soleus muscles of *Irf3^−/−^* and *Irf7^−/−^* mice. This was confirmed when we measured a reduced angiogenic capillary formation in the soleus muscles of *Irf3^−/−^* and *Irf7^−/−^*mice compared to C57BL/6 mice. The reduced number of capillaries was only observed in the left ischaemic calf and not in the right non‐ischaemic calf. This indicates that the genetic baseline in all mice was similar and the decreased ischaemia‐induced angiogenesis in the ischaemic muscles of *Irf3^−/−^* and *Irf7^−/−^*mice was purely based on the effects of IRF3 and IRF7. Previously, a role of IRF3 and IRF7 in angiogenesis was indicated. A phenotypic genome‐wide screening performed on human umbilical vein endothelial cell by Korherr et al identified factors that induce angiogenesis. Among these factors was the well‐known factor VEGF as well as TRIF, kinase TBK1 and IRF3.^28^ This indicates that the TRIF/TBK/IRF3 pathway has angiogenic properties. In addition, IRF7 is also involved in angiogenesis since Jin et al also showed that IRF7 over‐expression increased tumour formation with increased angiogenesis and cell heterogeneity by inducing inflammatory cytokine expression (IL6, CCL2 and CXCL1).^29^ This latter is in line with our results where we show an attenuated inflammatory response in absence of IRF7.

Since angiogenesis is induced via VEGF, it is interesting that we found a down‐regulation of VEGR2 in the ischaemic muscles of IRF3 and IRF7‐deficient mice. VEGF can bind to VEGR1, VEGFR2 and VEGFR3, but VEGFR2 is the main receptor transmitting VEGF signals.^30^ The lack of VEGFR2 in the ischaemic tissue can (partly) explain the decrease in angiogenesis in IRF3 and IRF7‐deficient mice compared to C57BL/6 mice. Interestingly, previously a relation between interferons, regulated by IRF3 and IRF7, and VEGF was observed. IFNs were able to mediate VEFG production from human mast cells^31^ and also in chronic myeloid leukaemia patients, imatinib in combination with pegylated‐IFN‐α2a was able to regulate VEGF levels.^32^

Arteriogenesis is induced by inflammation, which results in the maturation of pre‐existing collaterals into arterioles.^6^ For optimal neovascularization and thus post‐ischaemic blood flow recovery, both angiogenesis and arteriogenesis are essential.^21^ We observed reduced arteriogenesis in the adductor muscles of *Irf3^−/−^* and *Irf7^−/−^* mice compared to C57BL/6 mice. The number of arterioles was only increased in *Irf3^−/−^* mice compared to C57BL/6 mice. However, the diameter of the arterioles was significantly decreased in *Irf3^−/−^* and *Irf7^−/−^* mice compared to C57BL/6 mice, which is characteristic for arteriogenesis since this is defined as the maturation of pre‐existing collaterals.^21^ Interestingly, we showed a decrease in inflammatory cells around the arterioles and decreased mRNA expression of inflammatory cytokines *tnfα*, *ccl2* and *il6* in the gastrocnemius muscles. It is shown that several inflammatory cytokines and components are essential in the regulation of arteriogenesis such as TNFα,^33^ as well as the CCR7‐CCL19/CCL21 Axis,^7^ NK cells and CD4+ T cells^34^ and CD27‐CD70 T cell co‐stimulation.^25^ We previously demonstrated that IRF3 regulates pro‐inflammatory cytokines downstream TLR4 activation in vitro, in VSMCs and macrophages, prominent inflammatory cell types involved in arteriogenesis.^15^ Together with the diminished inflammatory response observed here, this partly explains how IRF3 and IRF7 affect neovascularization.

Although from the current data on cytokine expression and macrophage presence at 28 days, it cannot be excluded that one might also conclude that due to the reduced neovascularization response, inflammatory cytokine expression is reduced and not vice versa. Previous data from our and other groups have clearly shown that in early stage after induction of hind limb ischaemia hampered inflammatory responses lead to reduced neovascularization responses.^9^, ^10^, ^25^, ^34^

The regulation of pro‐inflammatory cytokines via IRF3 and IRF7, in general goes via the TRIF‐NFĸB pathway.^12^, ^35^ TRIF is also involved in TLR3‐mediated type‐I IFN production via IRF3 and IRF7 activation. It is suggested that activation of TLR3‐TRIF can also suppress angiogenesis via initiation of apoptotic cell death.^36^, ^37^, ^38^, ^39^ The ability of TRIF to induce apoptosis however, was not dependent on its ability to activate either IRF3 or NFĸB but was dependent on RIP, Fas‐associated death domain, caspase‐8‐dependent and mitochondrion‐independent pathway and the presence of an intact RIP homotypic interaction motif.^36^, ^37^ Thereby, it was suggested that TRIF‐IRF3‐IRF7 pathway activation, TRIF‐NFĸB pathway activation and apoptosis pathways are uncoupled and this can explain the differential functions of TLR3‐TRIF in neovascularization. Remarkably, we showed a regulation of pro‐inflammatory cytokines via IRF3 and IRF7, which indicates that there may be a linkage between the TRIF‐IRF and TRIF‐NFĸB pathways.

After phosphorylation downstream TLR3, IRF3 and IRF7 can produce type‐I IFNs, such as IFNβ. However, the role of type‐I IFNs and IRF3 and IRF7 in neovascularization is not identical. Schirmer et al observed attenuated post‐ischaemic blood flow recovery7 days after HLI in mice treated with type‐I IFN, IFNβ.^40^ In addition, blocking IFNβ demonstrated to stimulate arteriogenesis via VSMC proliferation^41^ and *Ifnar^−/−^* mice, deficient in the IFNβ receptor, improved blood flow recovery 7 days after induction of hind limb ischaemia. Teunissen et al observed that *Ldlr^−/−^* mice treated with monoclonal antibodies blocking IFN‐α/β receptor subunit 1 (IFNAR1) improved post‐ischaemic blood flow recovery via augmented arteriogenesis.^42^ These pre‐clinical results are seemingly in contrast with our results since we show an impaired blood flow recovery after HLI in *Irf3^−/− ^*and *Irf7^−/−^*mice. However, IRF3 and IRF7 do not only produce type‐I IFNs, such as IFNβ, but can also regulate pro‐inflammatory cytokine production, as we show in the gastrocnemius muscles of *Irf3^−/−^* and *Irf7^−/−^* mice. As we previously showed, TRIF‐ NFĸB‐mediated pro‐inflammatory cytokines are important regulators of neovascularization.^9^, ^10^, ^34^ In this respect, our results can only be partly explained by the effects of IFNβ and their IFNAR receptor. In addition, post‐ischaemic blood flow recovery was measured in the paw of the mice, which was used to determine arteriogenesis. Both angiogenesis and arteriogenesis are essential for accurate neovascularization and post‐ischaemic blood flow recovery.^21^ In addition to arteriogenesis, which we determined by measuring the diameter of the arterioles in the adductor muscles of the *Irf3^−/−^*, *Irf7^−/−^* and C57BL/6 mice, we also measured angiogenesis in the soleus muscles.

The findings of the present study indicate that deficiency of IRF3 and IRF7 results in impaired post‐ischaemic blood flow recovery caused by an attenuated angiogenesis and arteriogenesis linked to lack of inflammatory components in ischaemic tissue. In conclusion, IRF3 and IRF7 are essential regulators of neovascularization.

## 5 CONFLICT OF INTEREST STATEMENT

The authors confirm that there are no conflicts of interest.
