# Supplementary material for: IRF3 and IRF7 mediate neovascularization via inflammatory cytokines
Source: J Cell Mol Med. 2019 Apr 1;23(6):3888–96. doi: 10.1111/jcmm.14247 (PMC6533520; doi:10.1111/jcmm.14247)
Supplement: Supplementary file 1 [file JCMM-23-3888-s001.docx]

**Supplemental table and figures.**

**Table S1.**

|  | Control | *Irf3^-/-^* | *Irf7^-/-^* |
| --- | --- | --- | --- |
| Total number analysed | 6 | 9 | 7 |
| Sex | Male | Male | Male |
| Body weight t=0 | 28.16±0.76 | 27.01±0.31 | 28.01±0.34 |
| Body weight t=28 | 29.15±0.76 | 27.21±0.28 | 28.61±0.25 |

**Table S1.** Mice characteristics of *Irf3^-/-^*, *Irf7^-/-^* and control mice. Total number of analysed mice is shown (n=), as well as sex (male or female), body weight at day of the surgery (t=0) and at sacrifice (t=28). Body weight is shown as mean±SEM. Body weight at t=0 and t=28 was not significantly different in the *Irf3^-/-^* and *Irf7^-/-^* mice compared to control mice.

**Figure S1.**


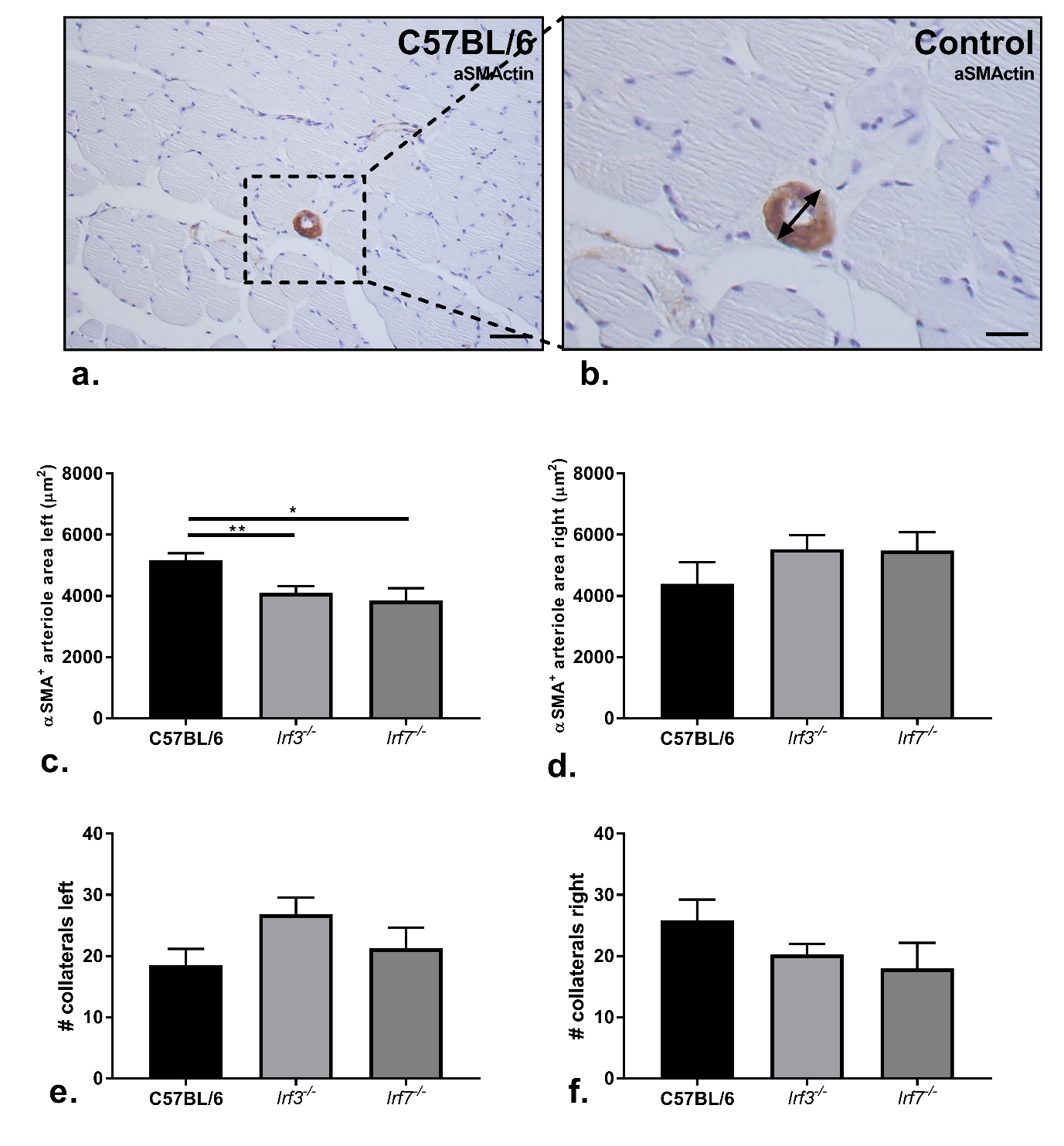


**Figure S1.** Arteriogenesis in aSMActin stained adductor muscles. **a.** Representative image of the left ischemic adductor muscle of control mice sacrificed 28 days after surgery, stained with aSMActin (20x magnification) and **b.** zoom in (40x magnification) with an arrow showing the way of measuring the diameter from outer membrane till outer membrane. **c.** αASMActin positive arterioles area of the left ischemic and **d.** right (non-ligated leg) adductor muscles is shown (µm^2^). Number of arterioles of the **e.** left ischemic and **f.** right non-ischemic adductor muscles stained with aSMActin is shown. Data is presented as mean SEM; * p<0.05; ** p<0.01, a 2-tailed Student’s t-test was used. Control n=6, *Irf3^-/-^* and *Irf7^-/-^* n=7.

**Figure S2.**


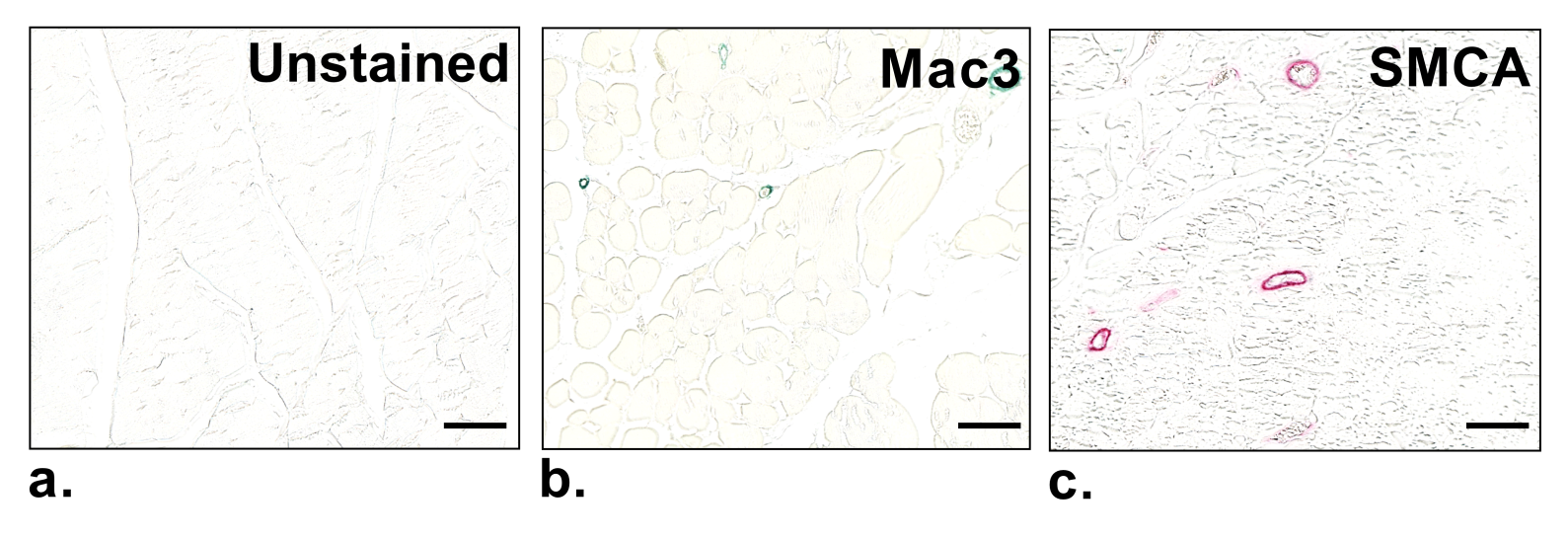
**Figure S2.** Inflammatory macrophages around collateral arterioles. An immunohistochemical double staining was performed on adductor muscles of *Irf3^-/-^*, *Irf7^-/-^* and control mice sacrificed 28 days after HLI. SMCA was used to show the collateral arterioles and MAC3 to show the macrophages around the collaterals. Representative image of **a.** an unstained adductor muscle. **b.** only MAC3 stained adductor muscle **c.** only SMCA stained adductor muscle is shown. (scale bar=50μm).

**Figure S3.**


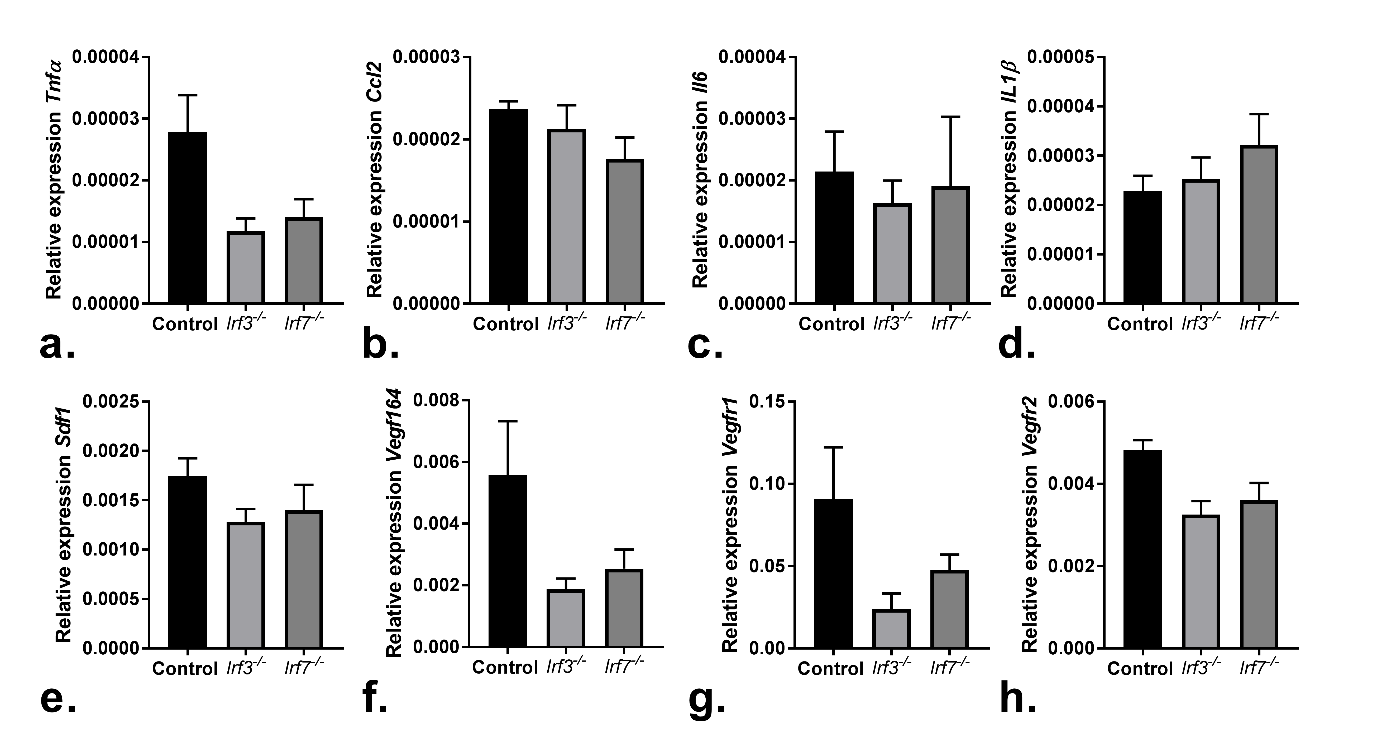


**Figure S3.** mRNA expression of essential genes for arteriogenesis and angiogenesis. RNA was isolated of gastrocnemius muscles of *Irf3^-/-^*, *Irf7^-/-^* and control mice sacrificed 28 days after HLI, and used for RT-qPCR analysis. Relative mRNA expression is shown of **a.** *tnfα*, **b.** *ccl2*, **c.** *il6*, **d.** *il1β*, **e.** *sdf1*, **f.** *vegf164*, **g.** *vegfr1*, and **h.** *vegr2*. Relative expression of mRNA is shown to GAPDH of the right gastrocnemius muscle (non-ligated leg). Data is presented as mean SEM, a 1-way ANOVA and a Kruskal-Wallis test was used. n=5-8.
